# All-Solution-Processable Robust Carbon Nanotube Photo-Thermoelectric Devices for Multi-Modal Inspection Applications

**DOI:** 10.3390/ma18214980

**Published:** 2025-10-31

**Authors:** Yukito Kon, Kohei Murakami, Junyu Jin, Mitsuki Kosaka, Hayato Hamashima, Miki Kubota, Leo Takai, Yukio Kawano, Kou Li

**Affiliations:** 1 Faculty of Science and Engineering, Chuo University, 1-13-27 Kasuga, Bunkyo-ku, Tokyo 112-8551, Japan; a21.6ta6@g.chuo-u.ac.jp (Y.K.); a21.ace5@g.chuo-u.ac.jp (K.M.); a24.stt5@g.chuo-u.ac.jp (J.J.); a21.gsta@g.chuo-u.ac.jp (M.K.); a21.ew7t@g.chuo-u.ac.jp (H.H.); a20.7c7e@g.chuo-u.ac.jp (M.K.); a20.ymc3@g.chuo-u.ac.jp (L.T.); 2 National Institute of Informatics, 2-1-2 Hitotsubashi, Chiyoda-ku, Tokyo 101-8430, Japan; 3 Kanagawa Institute of Industrial Science and Technology, 705-1 Imaizumi, Ebina-shi 243-0435, Kanagawa, Japan

**Keywords:** carbon nanotube, photo-thermoelectric effect, long-wavelength photo-monitoring, printable electronics, computer vision, non-destructive inspection

## Abstract

While recent industrial automation trends emphasize the importance of non-destructive inspection by material-identifying millimeter-wave, terahertz-wave, and infrared (MMW, THz, IR) monitoring, fundamental tools in these wavelength bands (such as sensors) are still immature. Although inorganic semiconductors serve as diverse sensors with well-established large-scale fine-processing fabrication, the use of those devices is insufficient for non-destructive monitoring due to the lack of photo-absorbent properties for such major materials in partial regions across MMW–IR wavelengths. To satisfy the inherent advantageous non-destructive MMW–IR material identification, ultrabroadband operation is indispensable for photo-sensors under compact structure, flexible designability, and sensitive performances. This review then introduces the recent advances of carbon nanotube film-based photo-thermoelectric imagers regarding usable and high-yield device fabrication techniques and scientific synergy among computer vision to collectively satisfy material identification with three-dimensional (3D) structure reconstruction. This review synergizes material science, printable electronics, high-yield fabrication, sensor devices, optical measurements, and imaging into guidelines as functional non-destructive inspection platforms. The motivation of this review is to introduce the recent scientific fusion of MMW–IR sensors with visible-light computer vision, and emphasize its significance (non-invasive material-identifying sub-millimeter-resolution 3D-reconstruction with 660 nm–1.15 mm-wavelength imagers at noise equivalent power within 100 pWHz^−1/2^) among the existing testing methods.

## 1. Introduction

Non-destructive inspection now plays an essential role in the recent fully automated industrial manufacturing and distribution sites. Among representative testing methods [1,2,3,4,5], large-area non-contact imaging via electromagnetic-wave facilitates effective inspection for its simultaneous acquisition of multiple points of information from target objects and the associated wavelength dependence as detectable behaviors. By shifting photo-irradiation wavelength ranges into millimeter-wave (MMW), terahertz-wave (THz), and infrared (IR) frequency bands, it is possible to non-intensively observe inner structures of objects opaque in visible light (Vis: human eyes) views [6,7,8,9,10]. Contrary to ultrahigh-resolution transmissive X-ray testing, MMW–IR imaging plays a complementary role for sensitively extracting metallic parts from non-metallic outers contained by the former in shielded environments and identification between metallic/non-metallic materials, or among non-metallic compositions themselves, by the latter in open conditions as pre-screening. Such advantageous non-invasive material identification via MMW–IR imaging is available by broadband photo-monitoring owing to specific optical properties (transmittance or absorptance) per targeted composition and irradiation wavelength, represented by spectroscopy such as ultraviolet (UV)-Vis-nearIR (NIR): UV-Vis-NIR, Fourier-transform IR (FTIR), and THz time-domain (THz-TDS) [11,12]. As typical spectrometers runs as bulky stational instruments, the development of functional photo-sensor materials is indispensable for upcoming on-site industrial non-destructive inspection applications [13,14,15] by satisfying ultrabroadband MMW–IR detection in high-sensitivity and thin, soft, and lightweight material configurations for flexibly designable compact systems per testing target.

As inorganic semiconductors or solid-state bulk chips play major constituents of the conventional, typical stational photo-detectors in MMW–IR regions [16,17,18,19,20], recent advances in solution-processable materials have garnered tremendous attention for the ease in fabricating them into thin, soft, and lightweight sensor devices at atmospheric room temperature conditions under normal light illumination without clean-room facilities. Some examples of solution-processable photo-sensor materials include Poly(3,4-EthyleneDiOxyThiophene)/Poly(4-StyreneSulfonate): PEDOT:PSS [21,22], multi-walled carbon nanotube: MWCNT [23,24], and so on. While PEDOT:PSS and MWCNT require external treatment (e.g., carrier doping or intercalation) to control their electronic states, single-walled CNT: SWCNT facilitates freely designable material properties by sorting its diameters as specific metallic–semiconducting mixture ratios. The related previous works have developed mass-production schemes for SWCNT as aqueous dispersions [25,26]; even manual handling processes are available, including suction filtration [27,28,29,30].

The use of SWCNT (just “CNT” from now on in this review) also exhibits other advantages for employing non-destructive MMW–IR image sensor (imager) together with the above solution-processable configurations. CNT provides ultrabroadband efficient photo-absorptance values across the entire MMW–IR region (further covering Vis ranges) as soft lightweight thin-film structures at a few micrometer thicknesses. The CNT-unique one-dimensional free carrier plasmon resonances delocalized in each constituent respective axial tube dominantly governs the above advantageous feature [31]. As CNT also exhibits sensitive thermoelectric conversion compared to that of metallic electrodes or other thin-films (e.g., PEDOT:PSS or MWCNT), such material properties facilitate the photo-sensor design under the uncooled non-vacuum ultrabroadband detection mechanism: photo-thermoelectric effect (PTE) [32]. The CNT film PTE MMW–IR imagers then have proved their advantageous presence [33,34,35,36,37,38,39,40] among typical existing photo-detectors, such as bolometers, high-frequency circuits, and plasmonic devices, for their optical performances (ultrabroadband sensitive sensing) and user-friendly mechanical configurations.

**Figure 1:**
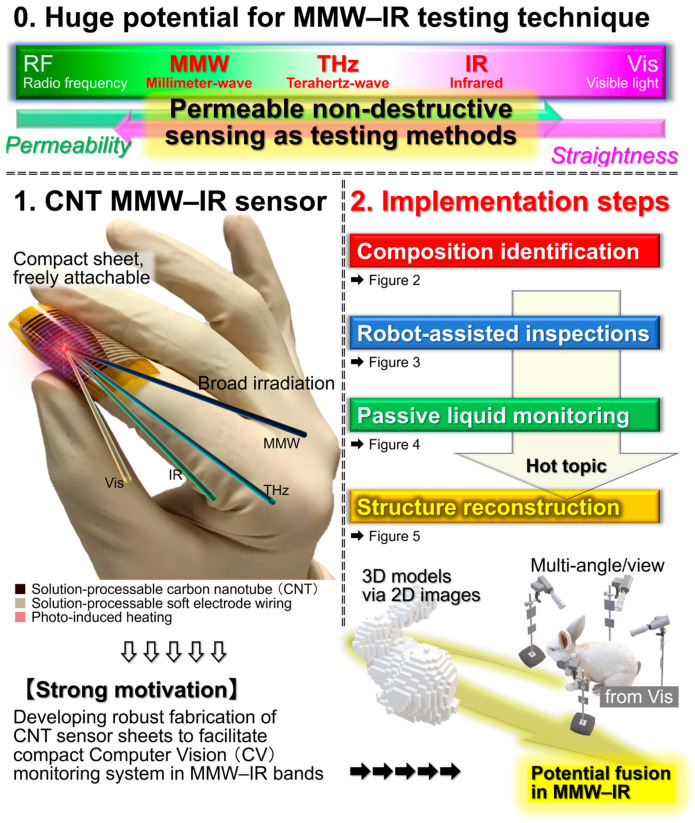
Conceptual aim of this review (reproduced from ref. [42] with permission).

**Figure 2:**
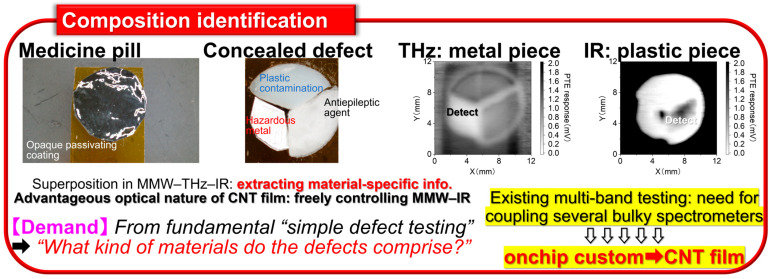
Testing platform “Compositional identification” based on the CNT film PTE MMW–IR sensor (reproduced from ref. [13] with permission).

**Figure 3:**
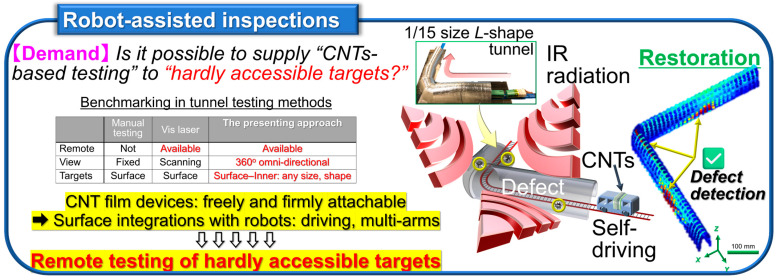
Testing platform “Robot-assisted inspections” based on the CNT film PTE MMW–IR sensor (reproduced from ref. [14] with permission).

**Figure 4:**
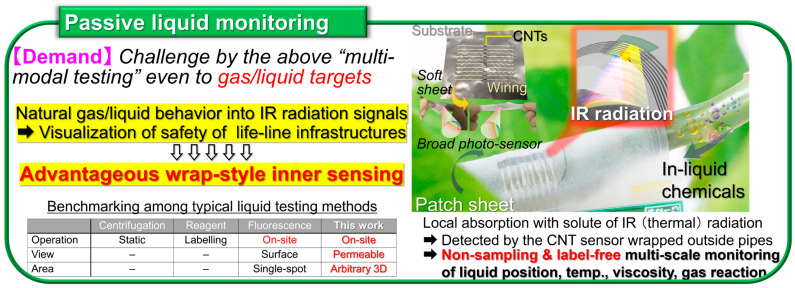
Testing platform “Passive liquid monitoring” based on the CNT film PTE MMW–IR sensor (reproduced from ref. [36] with permission).

**Figure 5:**
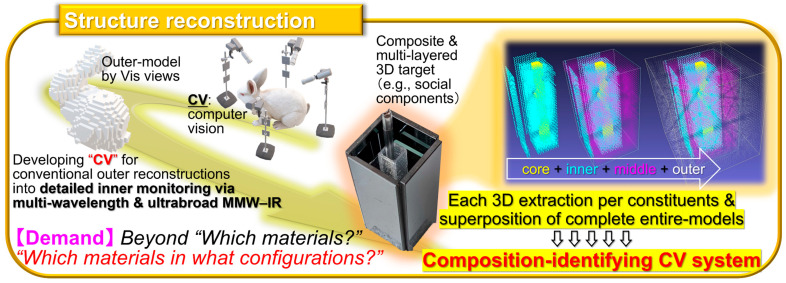
Testing platform “Structure reconstruction” based on the CNT film PTE MMW–IR sensor (reproduced from ref. [39] with permission).

To realize practical on-site non-destructive industrial inspection applications with the CNT film PTE MMW–IR imagers (Figure 1, Figure 2, Figure 3, Figure 4 and Figure 5), there are still two major issues remaining. One is to develop high-yield robust printable device fabrication processes by making the most of solution-processable configurations of CNT. The other one is to synergize non-destructive transmissive material composition identification by the CNT film PTE MMW–IR imagers with three-dimensional (3D) structural-reconstructing computer vision (CV). Since “electronics” have recognized printing as fast cost-effective fabrication [41,42,43,44,45], those approaches potentially play the key factor for practically implementing CNT film PTE MMW–IR imagers in the upcoming industrial testing where inspection accuracy relies on integration area and density of sensors. For the latter, CV typically refers to spatial information (e.g., coordinate, angle, distance) with inherent signals (e.g., intensity, wavelength, phase, time-delay) in optical measurements and converts these multi-scale assessments into 3D models. While CV has garnered tremendous attention in invasive X-ray testing or digital art platforms in Vis regions via computed tomography (CT), visual-hull (VH), and so on [46,47,48,49,50], the coupling with material-identifying MMW–IR monitoring is still insufficient. In other words, the development of high-yield robust fabrication of the CNT film PTE imager via printing processes potentially accelerates the synergetic effect as MMW–IR CV for under-friendly informative non-destructive industrial inspection. To this end, this review summarizes recent advances in the study field of CNT film PTE imagers with “screen-printing” as the high-yield robust device fabrication, and “MMW–IR VH” as the associated non-destructive inspection demonstration.

The motivation of this review is to introduce the above fusion of MMW–IR sensors with Vis-based CV as one of the hot scientific topics. Such a concept plays a functional role (non-invasive material-identifying sub-millimeter-resolution 3D-reconstruction at portable desktop systems with 660 nm–1.15 mm wavelength imagers at noise equivalent power within 100 pWHz^−1/2^) among the existing testing methods, including X-ray CT, Vis VH, IR LiDAR, and so on.

## 2. Device Mechanism

**Figure 6:**
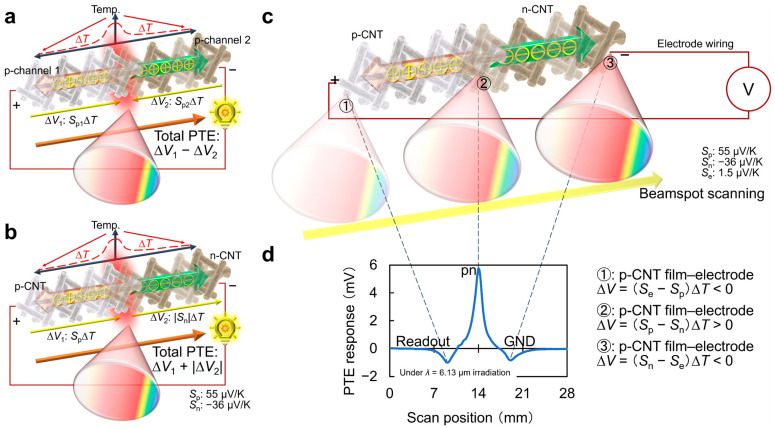
(**a**–**c**) Device model. (**d**) PTE response signal mapping (reproduced from ref. [39] with permission).

Figure 6a–c introduces the detailed device working mechanism of the CNT film PTE imager under external ultrabroadband (MMW–IR) photo-irradiation. The PTE synergizes two types of energy-harvesting phenomena as follows: (1) photo-absorption-induced heating at the CNT film channel and (2) subsequent TE conversion across the channel. This means that the device offers direct-current voltages as detection response signals of external photo-irradiation. The PET effect typically employs contact junctions as the photo-detection interface consisting of heterogeneous compositions for freely designing the effective Seebeck coefficient (*S*_eff_) in the channel. This is because the PTE response signal intensity against external photo-irradiation is proportional to *S*_eff_. and representative photo-detection interfaces employ channel-wiring electrode junctions. In other words, the use of sensor materials with large *S* enhances the photo-detection response signal intensity under the PTE. To design PTE sensor device structures, Ref. [39] in Figure 2 typically measures *S* of respective constituent materials with a consistent temperature difference of 5–6 °C across each channel (electrode and CNT films), for example. As *S* of metal is much lower than that of semiconductors, nanocarbon, and conductive polymers, the CNT film PTE imager employs its pn-junction, where the channel exhibits the maximum *S*_eff_, for the photo-detection interface (Figure 6d).

To employ the above device in optical imaging, the PTE further converts the photo-response signal intensity to monochrome color scale (e.g., higher and lower for brighter and darker). As the PTE response intensity at the pn-junction of the CNT film imager is larger than that of the remaining two channel-electrode interfaces by a magnitude over 5, the device also provides a single positive polarity signal even against full-face photo-irradiation. This means that the above imager is freely available not only for focusing single-point optical measurements but also large-area camera-like monitoring under full-face photo-irradiation.

## 3. Results

### 3.1. Screen-Printable CNT Film PTE Imager

#### 3.1.1. Fabrication Process

**Figure 7:**
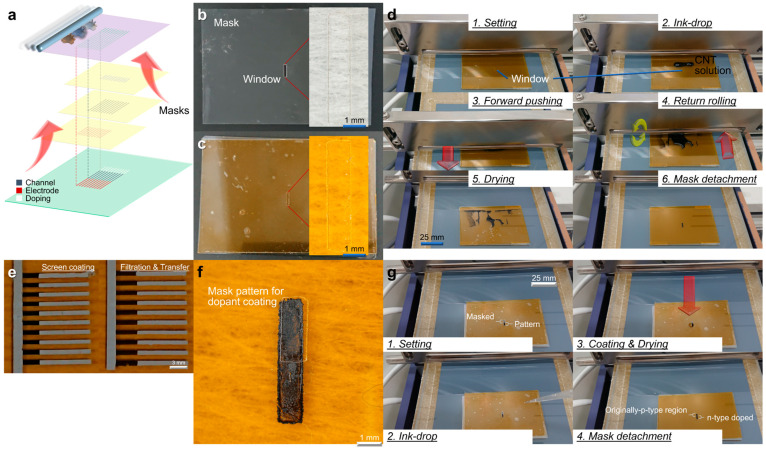
(**a**–**d**) Screen-printing of CNT. (**e**) Screen-printing of electrodes. (**f**,**g**) Screen-printing of dopants (reproduced from ref. [42] with permission).

The screen-printing shown in Figure 7a is a fabrication technique that realizes two-dimensional (2D) pixel integration with high density and subsequent camera device applications with high yield compared to conventional CNT film PTE imagers with low-yield operation. This process printed the material inks directly on the supporting substrate to design a pn-junction-type ultrabroadband PTE imager. The screen-printed CNT film PTE imager first required the preparation of a thin-film mask containing laser-processed windows using a desktop CO_2_ laser system (HAJIME CL1, Oh-Laser Co., Ltd., Kawagoe City, Japan) and attached it to the coating substrate with 25 μm thick polyimide (PI) tape (NO.760H, TERAOKA SEISAKUSHO Co., Ltd., Tokyo, Japan) (Figure 7b,c). The detailed experimental steps of the screen-printing were as follows: set the substrate on the glass supporter, firmly mounting the mask on the substrate, dropped ink materials on the windows, pushed the ink forward under high-pressure by the screen bar in “3. Forward pushing”, eliminated uneven coating by rotating backward and returning in “4. Return rolling”, dried, and detached the mask from the substrate.

Figure 7e shows an array PTE scanner based on screen-printing and filtration-transferred CNTs as channel-electrode ink (rubber-like readout electrode in ref. [41] comprised a silver powder-binder resin-mixed conductive paste (ELEPASTE NP1, TAIYO INK MFG Co., Ltd., Ranzan, Japan)) also fabricated by the former method. The printing accuracy of screen-printing is as high as that of filtration transfer. Ref. [41] easily formed a pn-junction by aqueous chemical-type electron-injecting dopants on half of the CNTs formed by screen-printing (Figure 7f,g). Regardless of the viscosity of the material inks, this process completed only screen-printing of the chemical dopant solution in a process similar to Figure 7d.

#### 3.1.2. Optical Property as Thin-Films

**Figure 8:**
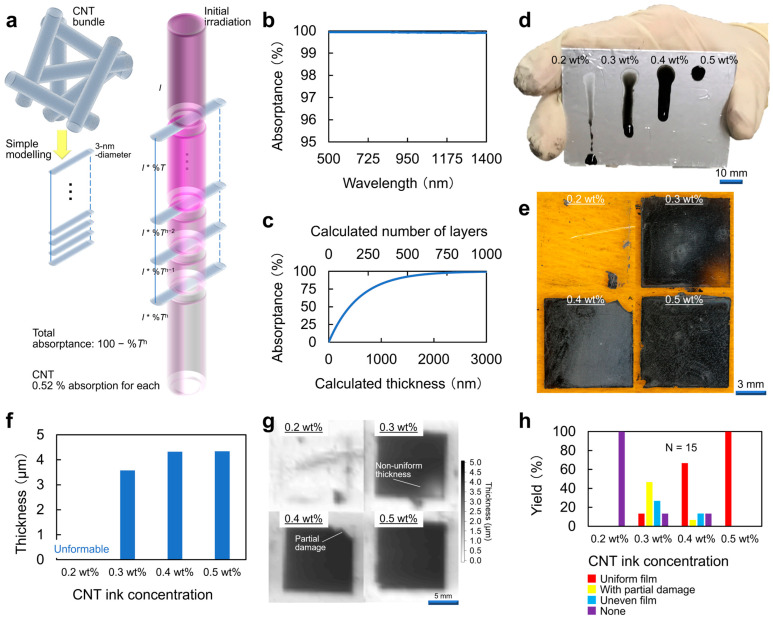
(**a**–**c**) Photo-absorption model of CNT. (**d**,**e**) Screen-printing of CNT. (**f**–**h**) Yield of screen-printed CNT films (reproduced from ref. [42] with permission).

Screen-printed CNT film exhibited an average photo-absorptance of approximately 99.9% across NIR to Vis bands. A simple structural model provides the relationship *A* = 100 − *T*^n^ to describe this multi-layer absorption behavior (Figure 8a). Figure 8b illustrates that a CNT film approximately 4 µm thick, consisting of around 1333 stacked CNT layers, provides near-complete absorptance of incident photo energy. Figure 8c shows the direct correlation between film thickness and the saturation of photo-absorptance. These results explained the need for a thickness of at least 3 µm to support effective photo-thermal conversion in CNT film PTE imagers.

Screen-printed CNT films for PTE imagers required careful control of the CNT ink concentration. Ink concentration significantly influenced the wettability on the substrate surface, resulting in film thickness and photo-absorptance. All these factors critically influenced the performance of CNT film. A diluter CNT solution had low viscosity and failed to coat the substrate uniformly. It did not effectively wet the surface (Figure 8d), so no continuous film formed (essentially zero yield) (Figure 8e). In other words, wettability and film-formability of inks improved as the CNT content increased, due to higher viscosity and solids loading. Increasing the CNT concentration to 0.3–0.4 wt% allowed deposition of a stable film a few micrometers thick (Figure 8f). Such 3–4 µm-thick screen-printed CNT films fulfilled the minimum thickness necessary for near-complete photo-absorptance across Vis and IR wavelengths. Notably, thicker films effectively captured longer IR wavelengths. Films From 0.3 to 0.4 wt% inks still exhibited partial non-uniform thickness and localized damage. Only a fraction of the films prepared with these concentrations formed completely uniform layers. Residual uneven areas or damage remained common. These imperfections indicated that high absorptance alone did not ensure optimally uniform or reliable device operation. Non-uniform regions in the CNT layer inevitably led to inconsistent PTE responses across different imager pixels.

At 0.5 wt% CNT concentration, screen-printing yielded a uniform, intact film without previous defects (Figure 8g). The higher viscosity of 0.5 wt% ink improved surface coverage during coating. Consequently, coating yield significantly increased, producing consistently usable films. The resulting 0.5 wt% ink density CNT film was approximately 5 µm thick. At this thickness, the film achieved nearly 100% absorptance of incident irradiation, fully exploiting the CNT layer’s photo-absorptive capability. Crucially, the increased CNT content at 0.5 wt% led to lower electrical resistance in the film. This reduced resistance directly suppressed thermal noise in the PTE sensor. Accordingly, CNT film imagers fabricated with 0.5 wt% ink exhibited the highest PTE sensitivity among all tested concentrations. In comparison, devices prepared with 0.3–0.4 wt% ink density films achieved similar absorptance levels because films were sufficiently thick to absorb most photo energy. However, low-concentration films still experienced slight thickness non-uniformity and higher electronic noise, undermining stable imager operation (Figure 8h). Overall, the 0.5 wt% ink density consistently produced reliable, high-performance CNT films. Optimal film formation, maximal absorptance and minimal electrical loss collectively delivered superior PTE-sensing performance. As ref. [41] unified the weight amount of in-liquid surfactant (Sodium line-ar-Alkylbenzenesulfonate) to 3 wt%, the obtained liquid viscosity trend in Figure 8 dominantly reflected the content of CNTs (single-walled, semiconducting-metallic unseparated, and edge-capped) for respective concentration inks.

#### 3.1.3. Stability and Robustness

**Figure 9:**
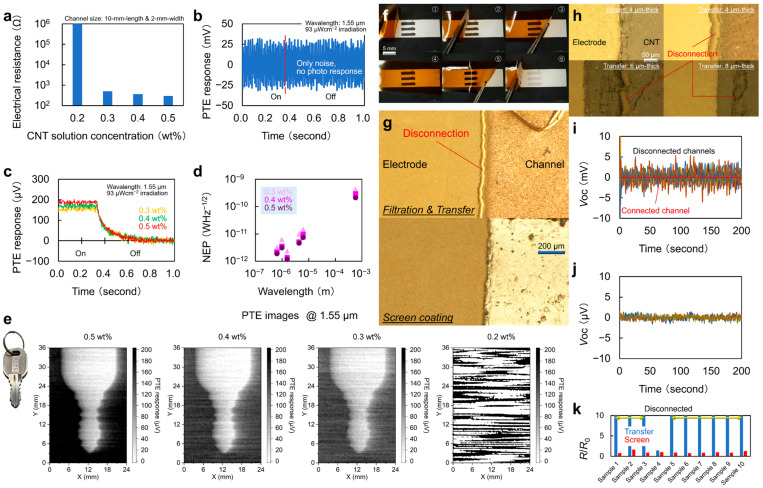
(**a**–**e**) Performances of the screen-printed CNT film PTE imager. (**f**–**k**) Thermophysical and electrical robustness of screen-printed CNT films (reproduced from ref. [42] with permission).

Ref. [41] provides the yield performance of screen-printed CNT films in relation to their concentration and demonstrates the superiority of the screen-printing method over other methods. Figure 9a exhibits the electrical resistance of screen-printed CNT films according to their weight concentration, and Figure 9b,c exhibits the photo-switching performance. Figure 9d exhibits the results of noise equivalent power (NEP). Increasing the CNT weight concentration reduces the NEP and improves the sensor performance. Figure 9e shows the IR imaging results of the key. As the CNT weight percentage increases, the image quality improves. A signal-to-noise ratio is 16.8 for the 0.3 wt% image, 18.3 for the 0.4 wt% image, and 27.5 for the 0.5 wt% image.

Figure 9f compares the adhesive transfer method and screen-printing technique. The adhesive transfer method deposits CNT film by using filtration on the PI tape. PI substrates are useful for PTE element design due to their high absorbance and ability to maintain heating in the sensor channel, which is beneficial for low thermal conductivity. The adhesive transfer method is a representative approach using tapes as a method for CNT film deposition on PI. Figure 9g exhibits a comparison adhesive transfer method with the screen-printing technique for CNT-silver paste interfaces. Ref. [41] utilizes a resin-mixed silver paste as an electrode, which undergoes volume shrinkage during annealing, thereby pulling adjacent CNT films. The screen-printing technique withstands pulling, as adhesion is strong to the substrate and CNT films. While the adhesive transfer method does not withstand pulling, as adhesion is fragile to the substrate and CNT films, which causes disconnection during annealing. As shown in Figure 9h, even with a change in film thickness, the adhesive transfer method exhibits a lack of annealing tolerance at the interface between silver paste and CNT. Figure 9i shows the open circuit voltage of 10 CNT film arrays implemented using the adhesive transfer method. It is clear that the open circuit voltage is high because it does not connect many elements. Figure 9j shows the open circuit voltage of 10 CNT film arrays implemented using the screen-printing technique. The screen-printing technique has a lower open circuit voltage and is conductive. Figure 9k shows a comparison of resistance values before and after silver annealing using the two methods. *R*_0_ is the resistance value measured after CNT deposition without applying silver paste. *R* is the resistance value after silver annealing. The smaller the *R*/*R*_0_, the better the deposition method. Therefore, screen-printing technique is an excellent deposition method with strong adhesion to the substrate.

#### 3.1.4. Chemical-Type Electron Injection

The fabrication of the CNT film PTE imager requires designing a pn-junction as the photo-detection interface. Ref. [41] employed the liquid coating chemical-type electron injection method to design pn-junctions in the pristine p-type CNT film channels. The chemical-type electron-injecting dopants consisted of potassium hydroxide (0.5 m KOH, Tokyo Chemical Industry Co., Ltd., Tokyo, Japan) and 15-crown 5-ether (C0859, Tokyo Chemical Industry Co., Ltd.). The crown ether selectively captures cation (K^+^), leaving anion (OH^−^) free. These free anions then inject electrons into the CNT films.

**Figure 10:**
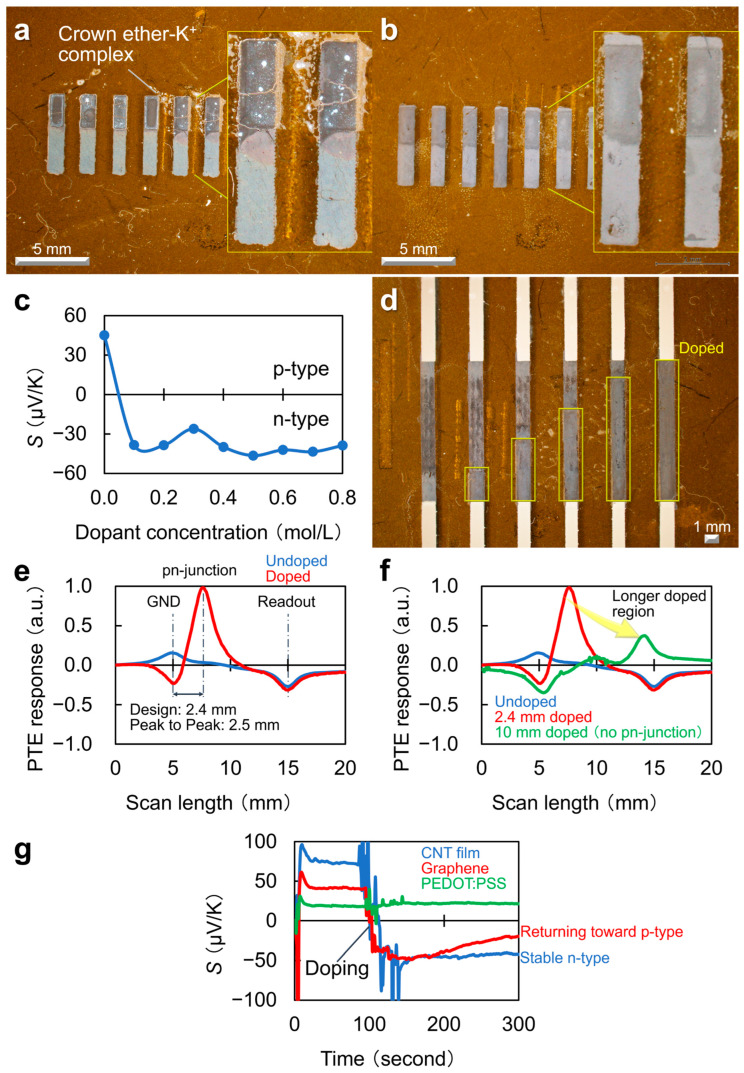
(**a**–**d**) n-type chemical carrier doping on CNT films via screen-printing. (**e**,**f**) Performances of the screen-printed pn-junction type CNT film PTE imager. (**g**) Material dependence (reproduced from ref. [42] with permission).

The chemically electron-injected region, i.e., screen-printed on half of the channel, is visually identifiable as shown in Figure 10a. This photograph captures the channel, including the electron-injected region, after temperature treatment at 120 °C for 10 min, corresponding to the electrode wiring condition. Note that the device fabrication involves screen-coating of chemical electron-injecting dopants before wiring of electrodes. This is because wiring electrodes adjacent to the CNT film potentially degrades the physical contact between the channel and the laser-processed window of the electron-injecting mask. The presented photograph shows the cracking that occurred in the electron-injected channel region. The screen-printing shown in Figure 10a utilized the electron-injecting dopant at 0.7 mol/L concentration. Figure 10b shows the screen-coating of electron-injecting dopants at the lower 0.1 mol/L concentration. In this case, it visually identifies no disconnections in the doped channel region. Based on these results, the excessive lamination of the complex from the dopant over the channel and its volumetric deformations (against thermal curing for electrode wiring) potentially induce the above disconnections. As the CNT film PTE imager fabrication includes electrode wiring after electron injection, the doped channel region must be robust for the temperature treatment of the electrically conducting paste.

Figure 10c illustrates the dependence of *S* on the concentration of chemical-type electron-injecting dopants. The graph in Figure 10b indicates that even the screen-coating of electron-injecting dopants at the concentration of 0.1 mol/L sufficiently converts *S* of the pristine p-type CNT film to a saturating negative coefficient. Thus, this condition satisfies the suitability of the screen-coating of electron-injecting dopants, mechanical robustness against temperature treatment for electrode wiring, and the fundamental applicability of the device as a pn-junction-type CNT film PTE imager.

Figure 10d demonstrates that the method enables high-precision control of the channel-doping region. Laser processing finely tunes the mask aperture size, which allows the process to incorporate a simplified mechanical alignment step into the dopant-deposition process. Figure 10e,f evaluates the mechanical alignment precision for the screen-coating of chemical-type electron-injecting dopants. The graphs introduce the line profiles of the PTE response via scanning a mid-IR (MIR) beam-spot (external photo-irradiation) along the device length direction. The CNT film PTE imager, consisting of the pristine p-type CNT channel (labeled “undoped” in the figure), exhibited local photo-responses in a reverse-polarity with double-peaks at both the ground- and readout-electrode interfaces. The CNT film PTE imager (electron-injected for the half channel area) offered single-peak mapping with the strongest response intensity at the pn-junction (as “Doped” in the figure). As the electron-injected channel was located on ground-electrode side in the device structure, the spatial gap in the mapping between the PTE response peaks at the pn-junction and the ground-electrode interface corresponds to the effective length of electron injection in the device. The proposed screen-coating method of electron-injecting dopants suppressed the spatial gap between the mask window design value (2.4 mm) and the effective doping length (2.5 mm) within 100 μm.

Figure 10g presents a graph comparing the effects of n-type dopants on representative conductive materials. The dopants employed in ref. [41] remained stable on CNT films under ambient air conditions at room temperature, highlighting greater stability compared with their behavior on graphene and PEDOT:PSS. These design principles provide a foundation for the high-yield fabrication of the CNT-film PTE imagers. This review also introduces the major specification of respective ink materials employed in ref. [41] for the experimental reference (Table 1).

### 3.2. Broadband Multi-Wavelength VH with the CNT Film PTE Imager

#### 3.2.1. System Setup

**Figure 11:**
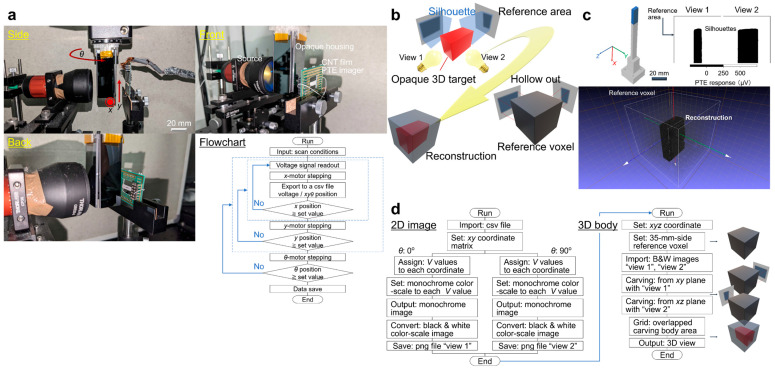
(**a**) Experimental setup. (**b**–**d**) Flowchart of VH (reproduced from ref. [39] with permission).

Figure 11a introduces the detailed setup with respective instruments for 2D-scanning and *θ*-rotation measurement, represented as a flowchart of the *xy* imaging. This system performs laser irradiation with four types of photo-sources: a frequency multiplier in the MMW band (Custom Modular Tx-Transmitter, Virginia Diodes Inc., Charlottesville, VA, USA), a CO_2_ gas laser in the THz band (L4, Access Laser Co., Everett, WA, USA), semiconducting laser fiber diodes in the IR (BL976-PAG900, Thorlabs, Inc., Newton, NJ, USA), and Vis (LP660-SF50, Thorlabs, Inc.) bands. This system employs beam expanders (Beam expanders, Thorlabs, Inc.) to expand the light of photo-sources. Ref. [39] operates spatial scanning using motorized digital stepping stages (Motorized Stage, Sigma Koki Co., Hidaka-shi, Japan) and employs a multiplexer data logger (34980A-34923A/T, KEYSIGHT TECHNOLOGIES Inc., Santa Rosa, CA, USA) to read out the signal. This system adopts LabVIEW (NI Co., Austin, TX, USA) for controlling these measurement devices, and Origin (OriginLab Co., Northampton, MA, USA) for acquisition of silhouette images.

Laser source, inspected object, and CNT thin PTE imager are in the same straight line in ref. [39]. The CNT film PTE imager detects the laser light that is transparent to the inspected object. The inspected object consists of the target to be extracted and opaque housing as an outer wall. Regarding the flowchart, ref. [39] operates *xyθ*-scanning of the inspected object. Firstly, ref. [39] operates *x*-scanning and reads out the PTE signal by defined x-step values. Then, when the *x* position surpasses the set *x* value, the inspected object returns to its original position on the *x*-axis. Secondly, ref. [39] operates the *y*-scanning. Where ref. [39] operates, *x*-scanning involves a series of movements per *y*-motor stepping. Then, when the y position surpasses the set value, the inspected object returns to its original position on the *y*-axis. Finally, ref. [39] operates *θ*-scanning. Similarly, ref. [39] operates *xy*-scanning, conducting series of movements per *θ*-motor stepping until the set *θ* value. A datalogger saves signal data after the measurement.

Figure 11b illustrates the operation flow for a simple 3D VH reconstruction. Ref. [39] considers cuboid structures as a target to be reconstructed, and the VH measurement employs two transmissive PTE silhouette images with different views at an angle gap of 90°. This system prepares a reference voxel to the size of the area applied by the *xy*-scan, and then hollows it from two different views at an angle gap of 90° using the PTE silhouette images acquired in advance. For “hollowing-out”, the overlapped bodies among the respective silhouettes within the reference voxel serve as the reconstructed structure.

Figure 11c provides the result of ref. [39] extracts the dominant opaque regions from the PTE silhouette images and then performs transmissive 3D VH reconstruction of an actual stereoscopically modeled target. The hollowing-out algorithm converts the PTE silhouette images from a monochrome gradation to a black-and-white color scale by setting a threshold value, half of the maximum transmissive PTE signal, to extract the dominant opaque regions. This system reconstructs the target shape by conducting back-projection to the reference voxel from extracted opaque regions on data processing tools like Python (version 3.10) and MeshLab (version 2021.10). The acquired 3D model offered a good agreement between the reconstructed views and the actual captured structural features of the original target appropriately. Figure 11d briefly describes data processing on 2D silhouette imaging in the following steps for 3D body reconstruction.

#### 3.2.2. Preparation of a Case Study Model

**Figure 12:**
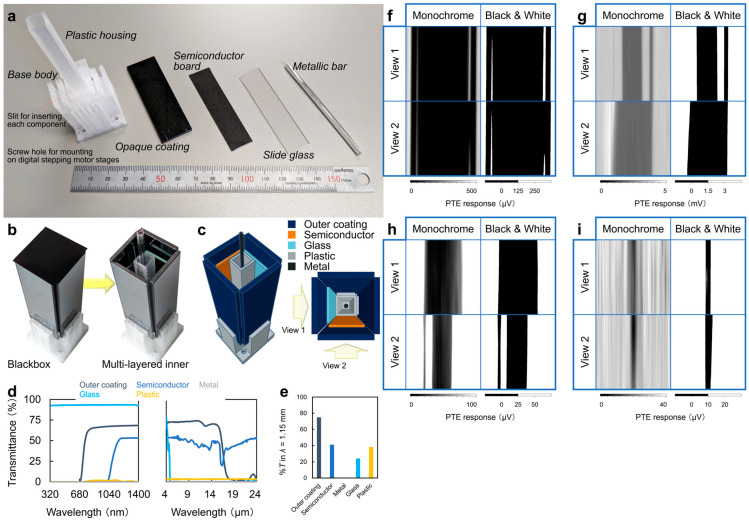
(**a**–**c**) Case study model. (**d**,**e**) Optical property of the model. (**f**–**i**) Transmissive 2D silhouette of the model by the CNT film PTE imager: (**f**) Vis, (**g**) IR, (**h**) THz, and (**i**) MMW (reproduced from ref. [39] with permission).

Figure 12a shows the materials used for the final structure of the composite multi-layered 3D object. The component materials in ref. [39] are the plastic housing, opaque coating, semiconductor substrate, slide glass, and metallic bar. Plastic housing is the foundation of the composite multi-layer cuboid object. The opaque coating is used not to visualize the object structure from the outside by human eyes. It inserts into the outermost layer base body of the plastic housing. The semiconductor substrate and slide glass compose the opaque coating on the inside. These materials are in a vertical position. The metallic bar is placed inside the center of the plastic housing, and it is cylindrical in shape.

Figure 12b is the final structure itself of the composite multi-layered 3D object. Structurally, opaque material firstly covers plastic housing. After that, semiconductor substrate and slide glass are set on the inside of the opaque material. In this way, it fabricates the middle layer and forms a multi-layered structure. Finally, the metallic bar is set the innermost layer of the plastic housing. Figure 12c is the schematic diagram of Figure 12b viewed from above and shows the relative position of the materials that correspond to View 1 and View 2. Note that View 1 and View 2 adopt as positioned references following experiment results.

Figure 12d,e are the materials evaluation of the optical characteristics in this experiment. Firstly, Figure 12d exhibits results obtained using UV-Vis and FTIR. The left one is the transmittance graph obtained from UV-Vis; the other is FTIR. Next, Figure 12e performs the transmittance graph of each material at MMW (1.15 mm). Ref. [39] uses lasers that are Vis (660 nm), IR (976 nm), THz (10.3 μm), and MMW (1.15 mm) for 3D structural reconstruction. Figure 12d,e shows that external MMW–IR-irradiation transmits the opaque material, so it is possible to evaluate the inside structure of the composite multi-layered target.

Figure 12f–i are images that compare the PTE silhouette of the composite multi-layered target. In order from Figure 12f–i, these are results of Vis, IR, THz and MMW. To obtain PTE silhouette images, it firstly irradiates laser lights from View 1 and View 2. The wavelength of laser lights is the same. After that, it restores the inside structure by gaining PTE response from the differences in transmittance. Figure 12f uses the laser of the wavelength (660 nm) that does not transmit the opaque coating of the outer layer, so View 1 and View 2 reconstructed silhouettes of the outer layer. The white slit in the figures is the gap when fabricating the composite multi-layered target. Figure 12g shows the results when it irradiated IR laser (976 nm). IR light transmitted the outer layer, and it non-destructively visualized the inner layer. View 1 captured the shape of the inner plastic housing that transmitted the slide glass of the middle layer. The right slit of plastic captures the semiconductor substrate of the middle layer, which is placed vertically. View 2 reconstructed the silhouette structure of the middle layer because IR light does not transmit the semiconductor substrate. Figure 12h is the results when it is irradiated to THz light. Figure 12h shows that THz light transmits the outer layer, and it visualized the inside. View 1 reconstructed the silhouette of the middle layer because THz light does not transmit the slide glass. View 2 captured the shape of the inner plastic housing that transmitted the semiconductor substrate of the middle layer. The left slit of plastic captures the slide glass of the middle layer, which is placed vertically. Figure 12i is the results when it is irradiated to MMW light. Figure 12i shows that MMW light transmits the outer layer, middle layer, and plastic, and it visualized the metal bar that is placed at the most inside. Ultrabroadband multiple-wavelength transmissive imaging measurements of the composite multi-layer object allow for the non-destructive extraction of each constituent structure due to the differences in the optical properties of the respective materials from these results.

#### 3.2.3. Non-Destructive Inspection Demonstration

**Figure 13:**
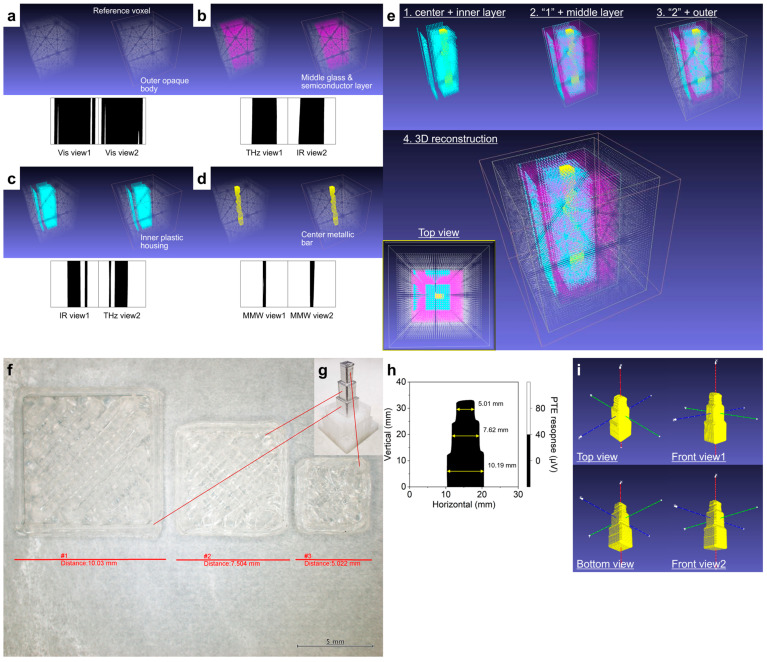
(**a**–**e**) Material-identifying 3D structure reconstruction of the case study model by the CNT film PTE imager. (**f**–**i**) Accuracy evaluation of the presented system (reproduced from ref. [39] with permission).

Ref. [39] demonstrated the non-destructive 3D reconstruction of the composite multi-layered objects by incorporating the visual hull approach and the obtained multi-wavelength 2D PTE images. Broad IR–MMW bands measurements effectively facilitate material identification for non-metallic substances through variable transmissive properties per wavelength and composition. Optical sensing-based computer vision techniques in the above bands potentially visualize the internal material layers within complex 3D structures in high resolution. The presenting CNT film PTE imager included in the system allows broad Vis–MMW bands operations according to the characteristic optical properties of the material. This advantage enables the 3D reconstruction of the composite multi-layered objects in broadband compared to existing computer vision techniques without switching the photo-detector across different wavelength ranges. Figure 13a clearly exhibits the capturing of the outer body in the original voxel using the 2D Vis PTE images rendered in a black-and-white color scale. Combined images of THz and IR bands provide the middle body model transferred with the slide glass and semiconductor board (Figure 13b) in opaque packaging (Figure 13a) and the plastic core shell (Figure 13c) in the middle layer region. As each IR and THz transparency to the object from “View 1” and “View 2” are complementary, the alternative utilization of the silhouette images in these wavelength bands selectively extracts the middle layer and inner bodies. The obtained MMW PTE images clearly visualized the metallic bar at the deepest center position of the composite multi-layered 3D object (Figure 13d). The integration of individually reconstructed models facilitates a multi-dimensional interpretation of the complex architecture of the composite multi-layered objects in the testing (Figure 13e) in a non-destructive monitoring manner. In the obtained 3D reconstruction model, the size and positioning of each constituent element of the target are in good agreement with the designed structure in Figure 12b,c. Ref. [39] defines the color and dot size of each 3D reconstruction model layer with the MeshLab coding. Additional approaches to increase the number of spatial viewpoints in the broadband visual hull provide further concrete shape estimations (cylinder, column, bumpy, curvature, and so on) for various different objects.

Ref. [39] verified the reconstruction accuracy of the visual hull monitoring technique with the CNT film PTE imager. This performance validation is essential for confirming the applicability of such methods in practical non-invasive inspection scenarios. The system demonstrated high reconstruction precision, enabling the accurate analysis of multi-layered composite structures in non-destructive testing. Figure 13f,g indicates a case study model for visual hull reconstruction. The width-lengths of the respective vertical layers are as follows: 10.03 mm (bottom), 7.504 mm (middle), and 5.022 mm (top). Figure 13h shows the black-and-white color scale PTE image of Figure 13g. The obtained results of the respective vertical layer widths are as follows: 10.19 mm (bottom), 7.62 mm (middle), and 5.01 mm (top). Ref. [39] finally calculated the error values of the presenting visual hull system as follows: 1.9% (bottom), 1.6% (middle), and 0.28% (top). Figure 13i provides the reconstructed 3D model of the stacked square pole. The CNT film PTE imager-based 3D visual hull reconstruction functions with a consistently high accuracy within 2% error. These results highlight the potential of the CNT film PTE imager to extract reliable structural information from opaque targets with comparable precision to existing computer vision techniques.

## 4. Discussion

Based on the above recent achievements, this review further summarizes major benchmarking (as a photo-sensor and as a CV technique) among related works, and potential upcoming scopes to advance the presence of the CNT film PTE imager.

As a photo-sensor, the CNT film PTE imager functions in ultrabroadband wavelength ranges covering 660 nm–6 cm without additional bulky antenna modules [32] with a minimum NEP of 560 fWHz^–1/2^ at atmospheric room temperature conditions [38]. Such functional features emphasizes the advantageous presence of the CNT film PTE imager among cutting-edge photo-sensor devices [51,52,53,54,55] toward practical non-destructive inspection applications. As a CV technique, the presented ultrabroadband VH also satisfies the fundamental system performances as follows: minimum spatial resolution of 650 µm, operation time of 150 s per photo-irradiation wavelength, and desktop module configuration, in addition to the aforementioned material-identifying 3D structure reconstruction and the size accuracy under an error ratio within 2%. Since such fundamental specifications of the presented system are well comparable to those of the existing narrowband CV [56,57,58,59,60], the achievements spotlighted in this review represent the significance of employing the CNT film PTE imager.

To further enrich these device and system performances, one of the potential upcoming scopes is to control nanoscale properties of respective constituent tubes for CNT films. As CNTs inherently exhibit optical selectivity per diameter [61,62,63,64,65], such nanoscale management of the PTE imager facilitates simultaneous ultrabroadband multiwavelength CV monitoring where respective photo-irradiation focus on the same single device. From another viewpoint, technical transition in the device fabrication further simplifies the CNT film PTE imager from the ongoing screen-printing with manual handling masks into all-mechanical-alignable air-jet dispensing or inkjet-patterning [66,67,68,69,70]. As such fine processing techniques (dispenser (hundreds of µm line width) and inkjet (tens of µm line width)) employ needle nozzles with a micrometer-scale air-gap diameter, the size management of in-liquid bundles for CNTs is essential. Commercialized CNT dispersions typically contain in-liquid bundles with diameters at hundreds of µm, and that size parameter dominantly governs the spatial resolution in integrating multiple-pixel CNT film PTE imagers. Dispenser and inkjet processing techniques of CNT dispersions also facilitate imager formation on supporting substrate materials with high thermal conductivity, accelerating the PTE conversion time constant under faster thermal diffusion. These approaches also facilitate multi-modal functional integration with other key sensing modules (e.g., humidity, chemicals, spatial acceleration, and so on) [71,72,73,74,75] or numerical-calibration-driven celestial sphere-view ball cameras [76,77,78,79,80] into freely attachable and repeatedly deformable thin-film configurations.

In addition to the above device fabrication strategy, optical coupling of the presented transmissive systems with reflective modules (e.g., light-detection and ranging: LiDAR) [81,82,83,84,85] further expands the identifiable material ranges from the aforementioned transparent/semi-transparent/opaque types into detailed transparent/reflective/absorbent configurations. The thin, soft, lightweight structure of the CNT film PTE imager also infers that the device setup is even available for high-speed augmented reality platforms [86,87,88,89,90] without interfering with their inherent complicated camera alignment systems.

## 5. Conclusions

**Figure 14:**
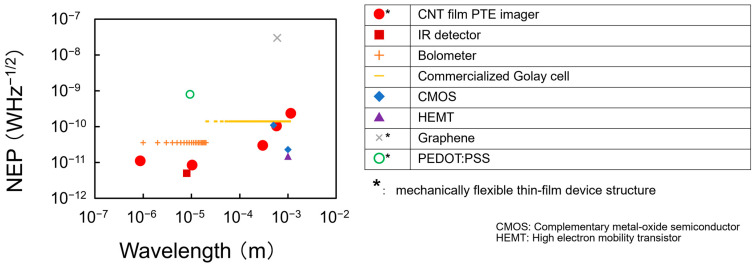
Benchmarking chart of the representative photo-detectors (reproduced from ref. [39] with permission). Respective references are as follows: CNT film PTE imager [14,39], IR detector [91], Bolometer [51], Golay cell [92], CMOS [52], HEMT [53], Graphene [54], and PEDOT:PSS [55].

In conclusion, this review summarizes several recent examples of the material and device design strategy for CNT film PTE imagers (as represented by promising performances among the existing photo-detectors in Figure 14 [14,39,51,52,53,54,55,91,92] with the sensor (1 mm-width and 5 mm-length for *R* of 555 Ω) [14]) with the associated scope for functional ultrabroadband non-destructive CV inspection.

**Figure 15:**
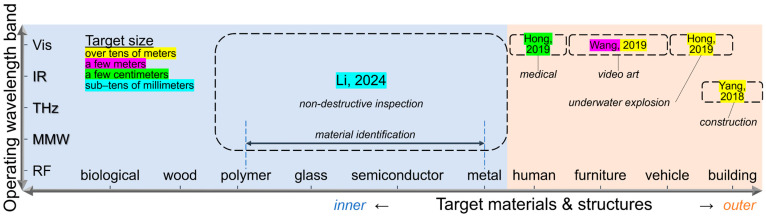
Benchmarking chart of the representative CV techniques (reproduced from ref. [39] with permission). Respective references are as follows: “*non-destructive inspection*” [39], “*medical*” [93], “*video art*” [94], “*underwater explosion*” [95], and “*construction*” [96].

This review also summarizes the major specification of representative CV techniques by Figure 15 [39,93,94,95,96] and Table 2 [39,50,58,60,85,95,97,98]. As PTE imagers facilitate ultrabroadband MMW–IR monitoring under flexible designable setups, CV in those regions plays an advantageous role among the conventional CT, LiDAR, and VH for safe, non-invasive operation and detailed material-identifying 3D structural reconstruction. Ref. [49] also reports further advancement for ultrabroadband MMW–IR 3D CV with hybrid algorithms between VH (opaque silhouette) and CT (semi-transparent) by making the most of the compact and sensitive usability for the CNT film PTE imager within the single transmissive experimental setup. Such hybrid MMW–IR VH advantageously performs effective non-destructive inspection within a few minutes by selective detailed CT for extracted body areas of targets via simple pre-VH-screening, while entire 3D tomography typically requires tens of hours. These fusions between PTE material science and ultrabroadband MMW–IR CV contribute to practical industrial non-destructive applications [99] via the upcoming large-scale social implementation.

## Figures and Tables

**Table 1:** Major specification of respective ink materials for the screen-printed pn-junction type CNT film PTE imager.

Specification	“CNT”	“Electrode”	“n-Dopant”
1. Solute	Single-walled semiconducting-metallic unseparated CNT	Silver particle and acrylic resin	Crown-Ether and Potassium Hydroxide
2. Solvent	Deionized water	Organic chemical	Deionized water
3. Surfactant	Sodium linear-Alkylbenzenesulfonate	—	—
Mixture ratio of 1–3	1 (0.2 wt%), 2 (over 97 wt%), 3 (3 wt%)	1 (71%) 2 (29%)	1 (12%) 2 (88%)

**Table 2:** Major specification of the representative CV techniques (method: including inner structures/reproduced from ref. [39] with permission).

Method	Wavelength Band	Accuracy and Resolution	Operation Time	System Size	Safety	System Cost	Operating Condition	Target & Use
MMW–IR–Vis VH [39]	660 nm –1.15 mm	Within 2% error	150 s per layer	Desktop	Non- invasive	20 k$	Indoor (ubiquitous)	Non-metallic outer–inner, Metallic inner
		650 µm						Material identification
Vis visual- Hull [95]	Vis	Within 3% error	0.1 s per picture	Vehicle module	Non- invasive	Over 1 M$	Outdoor	Construction
		54 cm						Outer-shape observation
LiDAR [60]	1550 nm	N.A.	20 milli s	Desktop	Non- invasive	15 k$	Outdoor	Human
		3.75 cm						Motion capture
sub-THz LiDAR [85]	3 mm	N.A.	130 s per picture	Meters-size desk	Non- invasive	10 k$	Indoor (in dried air)	Cardboards, metals
		1 cm						Identifying concealed metal cans
X-ray CT [97]	X-ray	Within 0.005% error	Several hours	Room-size	Invasive	Over 1 M$	Shielded	Bio-samples, Non-metallic outer–inner, Metallic inner
		10 µm						Inner imaging, Bio-imaging
IR CT [98]	1250 nm	N.A.	0.2 s	Room-size	Non- invasive	Over 1 M$	Shielded	Mice
		50 µm						Heart-shadow imaging
THz CT [58]	150 µm	N.A.	6 min	Meters-size desk	Non- invasive	100 k$	Indoor (in dried air)	Plastic
		1 mm						Inner metal imaging
Photo- acoustic [50]	Vis	Within 2% error	10 s	Meters-size desk	Non- invasive	100 k$	Indoor (dark room)	Only bio-samples
		370 µm						Bio-imaging

## Data Availability

No new data were created or analyzed in this study. Data sharing is not applicable.
